# Post-event Processing Predicts Impaired Cortisol Recovery Following Social Stressor: The Moderating Role of Social Anxiety

**DOI:** 10.3389/fpsyg.2017.01919

**Published:** 2017-10-31

**Authors:** Shunta Maeda, Tomoya Sato, Hironori Shimada, Hideki Tsumura

**Affiliations:** ^1^Graduate School of Human Sciences, Waseda University, Tokorozawa, Japan; ^2^Research Fellow of Japan Society for the Promotion of Science, Tokyo, Japan; ^3^Institute of Humanities, Social Sciences and Education, Niigata University, Niigata, Japan; ^4^Faculty of Human Sciences, Waseda University, Tokorozawa, Japan; ^5^Department of Environmental Medicine and Public Health, Shimane University, Izumo, Japan

**Keywords:** social anxiety, salivary cortisol, post-event processing, stress, recovery, TSST

## Abstract

There is growing evidence that individuals with social anxiety show impaired cortisol recovery after experiencing social evaluative stressors. Yet, little is known regarding the cognitive processes underlying such impaired cortisol recovery. The present study examined the effect of post-event processing (PEP), referred to as repetitive thinking about social situations, on cortisol recovery following a social stressor. Forty-two non-clinical university students (23 women, 19 men, mean age = 22.0 ± 2.0 years) completed the Trier Social Stress Test (TSST), followed by a thought sampling procedure which assessed the frequency of PEP reflecting the TSST. A growth curve model showed PEP and social anxiety interactively predicted cortisol recovery. In particular, PEP predicted impaired cortisol recovery in those with low levels of social anxiety but not in those with high levels of social anxiety, which contradicted the initial hypothesis. These findings suggest that PEP is differentially associated with cortisol recovery depending on levels of social anxiety. The possible mechanisms underlying these findings were discussed in terms of protective inhibition framework.

## Introduction

Social anxiety is characterized by marked fear of being scrutinized during social interactions (American Psychiatric Association, 2013). Recently, altered hypothalamic-pituitary-adrenal (HPA) axis responses to psychological stressors have been implicated in social anxiety (e.g., Furlan et al., 2001; Condren et al., 2002; Shirotsuki et al., 2009; Elzinga et al., 2010). Although the directionality of these alterations are not fully known, a recent meta-analysis revealed that individuals with social anxiety disorder (SAD) show heightened cortisol responses to psychological stressors, and this effect is most prominent during recovery periods (more than 25 min post-stressor offset; Maeda et al., 2016). Generally, HPA-axis dysregulation is associated with social avoidance behaviors (e.g., Kalin et al., 1998). Indeed, previous findings suggest that cortisol responses can facilitate the avoidance of socially threatening stimuli among individuals with SAD (Roelofs et al., 2009; van Peer et al., 2009). Such avoidance behaviors can prevent individuals from habituation to socially threatening situations, which lead to persistent fear responses. Thus, impaired cortisol recovery following social evaluative stressors likely has a crucial role in the psychopathology of social anxiety.

Although previous studies have examined cortisol responses among socially anxious individuals, there is few study that has directly examined potential mechanisms underlying impaired cortisol recovery within this population. To this end, cognitive models of social anxiety are useful. Several models have provided descriptions for the maintenance of social anxiety (e.g., Clark and Wells, 1995; Hofmann, 2007), where information processing characteristic of social anxiety is considered to prevent individuals from incorporating adequate social information. One example of information processing is the post-event processing (PEP). PEP refers to repetitively thinking about social situations even after leaving or escaping those situations (Clark and Wells, 1995). PEP can lead to prolonged processing of social-evaluative threat, which may be a key mechanism underlying impaired cortisol recovery observed among socially anxious individuals. Indeed, a few previous studies examined the effect of post-stress thoughts associated with PEP on cortisol recovery. For example, Zoccola et al. (2008) showed that PEP assessed 10 min after a speech task using a self-report questionnaire was associated with impaired cortisol recovery. On the other hand, Shull et al. (2016) examined the effect of experimentally induced post-stress thoughts focusing on negative evaluation by judges in stress testing, but found no effect on cortisol recovery. Thus, although methodological differences exist among studies, findings are not consistent about whether PEP is associated with impaired cortisol recovery.

One possible explanation for this inconsistency is that the effect of PEP differs among individuals. That is, according to the cognitive model of social anxiety, reviews of social interactions are likely to be threatening, particularly among individuals demonstrating social anxiety symptoms, because they hold strongly to the negative images (Clark and Wells, 1995). However, to our knowledge, there is no study which examined the effects of both PEP and social anxiety on cortisol recovery.

Therefore, the present study examined the effect of PEP on cortisol recovery in individuals with high and low levels of social anxiety. We hypothesized that PEP would be associated with impaired cortisol recovery. Additionally, the effect of PEP should be more prominent for those reporting higher levels of social anxiety than those with lower levels.

## Materials and methods

### Study participants

Forty-two Japanese university students participated in this study (23 women, 19 men, Mean age = 22.0 years, *SD* = 2.0). Participants were recruited through advertisements on the university campus. In the advertisement of the study, participants were informed that the experiment includes an “interview” before a panel, but no further details of the task were given. Individuals were deemed ineligible if they met any of following criteria: (a) history of a diagnosed psychiatric disorder, (b) stressful experiences just prior to the experiment, (c) history of smoking, (d) use of medications that could affect cortisol responses (e.g., oral contraceptives, β-blockers), (e) suffering from severe sleep disturbance or fatigue, (f) and irregular menstruation (for women). Women also provided menstrual phase information on the day of the experiment. Participants were asked to refrain from consuming alcohol and caffeine on the day of the experiment. In addition, they were asked to refrain engaging in vigorous exercise and consuming food for 1 h prior to participation in the study. All participants provided written informed consent and were told they could withdraw from the study at any time. Participants received a book coupon worth ¥2000 (approximately $18) as compensation for their participation. The study was approved by the Waseda University Academic Research Ethical Review Committee, and all participants provided written informed consent.

### Procedure

All testing was performed between 2 and 7 pm to control for circadian variation in cortisol activity. We used a standard acute psychosocial stress test, the Trier Social Stress Test (TSST), in which participants were required to deliver a speech and perform mental arithmetic in front of two audiences (Kirschbaum et al., 1993). The TSST has been repeatedly used to examine cortisol responses in the context of social anxiety (e.g., Roelofs et al., 2009; Shirotsuki et al., 2009; Elzinga et al., 2010).

At the beginning of the experiment, participants provided written informed consent. Next, participants completed psychological assessment questionnaires. These questionnaires took approximately 10 min to complete. Participants then remained seated in a quiet room for 10 min to control for any potential confounds prior to initial cortisol sampling. After the baseline assessment, participants were given TSST instructions. After preparing for a public speech for 10 min, participants delivered the speech for 5 min and performed a mental arithmetic task for 5 min. Following the TSST, participants performed the cognitive tests as part of the PEP assessment, which lasted for approximately 40 min. Participants took an additional 10 min to rest after the cognitive tests and finished the experiment. Throughout the testing period, participants refrained from eating and drinking anything but little water.

Saliva collection and assessment of state anxiety were conducted at eight time points: baseline, after speech preparation, just after the TSST, after each block of the cognitive tests, and after a 10-min rest following cognitive tests. These assessments largely corresponded to the following time periods with respect to TSST offset: −20, −10, 0, +10, +20, +30, +40, +50 min.

### Measures

#### Psychological assessments

Levels of social anxiety were assessed with two measures: the Social Phobia Scale (SPS; Mattick and Clarke, 1998) and the Social Interaction Anxiety Scale (SIAS; Mattick and Clarke, 1998). These measures assess fear associated with performing in public and specific social interactions, respectively. Both consist of 20 items that are rated on a five-point Likert-type scale (range: 0–80). SPS and SIAS were translated into Japanese and validated by Kanai et al. (2004). The SPS and the SIAS scores were standardized and averaged to form a social anxiety composite score (SA composite, hereafter). Additionally, we assessed levels of depression using the Center for Epidemiologic Studies Depression Scale (CES-D; Radloff, 1977). The CES-D is a self-report measure designed to assess depressive symptomatology within the general population and consists of 20 items that are rated on a four-point Likert-type scale (range: 0–60). The CES-D was translated into Japanese and validated by Shima et al. (1985). We also assessed subjective state anxiety during the experiment using a Visual Analog Scale (VAS) immediately before each saliva sampling. Anchor values of zero and 100 were defined as “not at all” and “extremely” anxious, respectively.

#### Post-stress thought sampling

Participants performed two rounds of two cognitive tests in counterbalanced order: a choice reaction task (CRT) and a working memory task (WMT). This paradigm has been used routinely in previous studies that deal with self-generated thoughts (e.g., Smallwood et al., 2011). Indeed, this paradigm has been applied to assess self-generated thoughts subsequent to the TSST (Engert et al., 2014). Thus, we applied this paradigm in order to assess PEP in the post-stressor period. During the CRT, participants observed sequences of white digits displayed on a black background on a computer screen while waiting for a red-colored digit, at which point participants had to indicate the parity of this target (odd or even) with a button press. During the WMT, participants were exposed to a sequence of white digits and intermittently probed with a red-colored question mark (“?”). When the question mark was presented, participants had to indicate the parity of the previous digit with a button press. For both tasks, white digits were presented for 1,000 ms, and colored stimuli were presented for 2,000 ms. Events were separated by a fixation cross at a random duration (2,200, 2,800, 3,200, or 4,400 ms). Targets (or question marks) and non-targets were presented at a ratio of approximately 1/6. There were a total of 21 targets during both the CRT and the WM tasks.

During both tasks, PEP occurrences were recorded using thought-probes method. Intermittently during the task, participants were interrupted with questions, and they responded “Yes/No” via a button press. Since PEP involves two aspects—thoughts about negative social events and cognitive interference (e.g., Wong, 2015)—we used two questions representative of these components: “Were you thinking about negative things that occurred during the interview task just before?” and “Were your thoughts about the interview task interfering with your concentration just before?” These questions were developed based on the PEP Questionnaire-Revised (McEvoy and Kingsep, 2006). In total, 12 probes for these two questions were presented during the tasks. Individuals' levels of PEP were defined as the number of “Yes” responses to the questions during both tasks (range: 0–24).

#### Cortisol levels

Participants were asked to draw saliva from their mouth for 2 min and drool into a specimen tube through a 4-cm long straw (passive drool). Saliva samples were frozen in a freezer at temperatures below −20°C until assay. Salivary cortisol levels were measured by means of enzyme-linked immunoassay using a commercial kit from Salimetrics (State College, PA, USA). The inter-assay variability was 7.0% (below 15.0% is generally acceptable).

#### Statistical analyses

To ensure that the TSST successfully served as a social evaluative stressor, we conducted a one-way repeated measures ANOVA with time and subjective anxiety. For cortisol values, we examined the impact of PEP and social anxiety on cortisol recovery using a two-piece multilevel growth-curve model with landmark registration (GCM-LR; Lopez-Duran et al., 2014). This model enables simultaneous modeling of post-stress peak, rise toward peak (activation), and decrease from peak (recovery) in cortisol. At the same time, this model controls for baseline levels and individual differences in the timing of peak cortisol. Furthermore, this model is found to be more sensitive than traditional statistical approaches in identifying group differences in cortisol trajectories (e.g., repeated-measures ANOVA and area under the curve; for details see Lopez-Duran et al., 2014).

First, individual post-stress peaks are identified from a visual analysis of the individual curves using Lopez-Duran et al.'s (2014) peak identification procedure and formula. To this end, each participant's response curve was visually inspected, and peaks were defined as the first point in the activation slope that was at least 15.5% greater than the baseline, which was followed either by a plateau or a decline. This 15.5% criterion has been shown to effectively distinguish cortisol responders and non-responders (Miller et al., 2013). If a plateau followed the peak, none of the samples in the plateau could be more than 10% higher than the peak; otherwise, the higher sample was considered the peak. Participants with peaks that met the above definition were labeled “responders” while those without peaks were labeled “non-responders.” Second, the timing (minutes from TSST offset) of each individual peak was identified (PeakTime) and was used to create a new time variable reflecting minutes from peak (MinFromPeak) using the following formula:

$$\begin{array}{l} \text{MinFromPeak }=\;\left(\text{PeakTime }-\text{ Time}\right)\;\times \;\left(-1\right) \end{array}$$

where Time is the minutes from the offset of the TSST. This new variable essentially adjusts all curves so that the peak for each individual falls on the same value (MinfromPeak = 0). For those without an identifiable peak (i.e., non-responders), we used the +10 time point (the mode peak time) as their expected, but not observed, “peak time” in order to model their non-response. Finally, we created two time variables to represent minutes before (TimeBeforePeak) and after the peak (TimeAfterPeak) using the following formulas:

$$\begin{array}{lll} \text{IF MinfromPeak } & < & \;0\text{ then TimeBeforePeak}=\text{MinfromPeak}\\ \text{Else MinfromPeak }=\;0.\\ \text{IF MinfromPeak } & > & \;0\text{ then TimeAfterPeak}=\text{MinfromPeak}\\ \text{Else MinfromPeak }=\;0. \end{array}$$

We then conducted a multilevel random effects model of the pre- and post-peak cortisol trajectory with peak levels as the intercept. The unconditional fixed effects model was defined as:

$$\begin{array}{lll} \text{Cortisol} & = & \beta_{0}\;+\;\left(\beta_{1}\;\times \text{ TimeBeforePeak}\right)\\ +\;\left(\beta_{2}\;\times \text{ TimeAfterPeak}\right)\;+\text{ e} \end{array}$$

where β_0_ is the intercept (peak), β_1_ is the activation slope, and β_2_ is the recovery slope. All models included random intercepts while controlling for cortisol baseline levels. Additionally, since previous studies have demonstrated that gender and depression potentially affect cortisol values (Burke et al., 2005; Kajantie and Phillips, 2006), we included gender and depression as covariates. All cortisol samples were box-transformed to normalize their distribution using the following formula (Miller and Plessow, 2013):

$$\begin{array}{l} {\text{X}}^{\prime }\;=\;\left({\text{X}}^{0.26}\;-\;1\right)/0.26. \end{array}$$

We also calculated effect size (Hedges' *g*) for significant predictors. Hedges' *g* was calculated based on conversion of *t* values produced by modeling using the equation of Rosnow et al. (2000) (see Floman et al., 2017).

In addition to these primary analyses, we provided scatterplots of the relationship between time and cortisol for low and high social anxiety and PEP based on median split (Supplementary Figure 1). We also provided similar scatterplots of the relationship between time and state anxiety (Supplementary Figure 2). Further, we examined whether cognitive task performance was affected by social anxiety and PEP with hierarchical regression analysis (Supplementary Table 1). These supplementary materials can be found in Data Sheet 1 (see Supplementary Material).

## Results

### Sample characteristics

Descriptive statistics (mean and standard deviations) for demographic information and self-report questionnaires are presented in Table 1. As for the levels of social anxiety symptoms in the present sample, the mean values of SPS and SIAS in Japanese non-clinical population examined in the previous study were 19.10 and 30.08, respectively (Kanai et al., 2004). The mean values of SPS and SIAS in the present sample are slightly higher than those in the previous study, but are much lower than those of the clinical population in Japan (SPS: 32.30, SIAS: 47.70). Most female respondents participated during the late luteal or early follicular phase of their menstrual cycle, during which influence of sex hormones on HPA axis is minimized (early follicular phase: 26.1%, late follicular phase: 13.0%, late luteal phase: 60.9%, according to coding criteria for in Duffy et al., 2017). The overall cortisol response rate was 71.4%, which was almost comparable to the response rates in previous studies (e.g., >70%; Kirschbaum et al., 1993). In addition, a one-way repeated measures ANOVA on subjective anxiety revealed a significant effect of time, *F*_(7, 287)_ = 52.64, *p* < 0.001. After correcting for multiple comparisons, results showed that that participants exhibited elevated anxiety in anticipation of the TSST (at −10 min; *p* < 0.001), which lasted even after they completed the TSST (at 0 min; *p* = 0.04). Taken together, the TSST successfully operated as a socially evaluative stressor.

**Table 1:** Descriptive statistics for demographic information and self-report questionnaires (*N* = 42).

	**Mean**	**SD**	**Max**	**Min**
Distribution of gender (women %)	54.8	–	–	–
Age	22.0	2.0	28	20
SPS	21.26	13.12	61	4
SIAS	34.74	13.75	72	10
CES-D	15.19	11.33	50	2

*SPS, Social Phobia Scale; SIAS, Social Interaction Anxiety Scale; CES-D, Center for Epidemiologic Depression scale*.

### Unconditional model of cortisol responses

In order to confirm that expected rise and fall in cortisol levels occurred in response to the TSST, we examined the unconditional model of cortisol trajectory, where no level-2 predictors were included in the model. Salivary cortisol levels significantly increased from baseline (Activation Slope; *b* = 0.010, *p* < 0.001) and declined significantly after reaching their peak (Recovery Slope; *b* = −0.009, *p* < 0.001). Thus, the expected rise and fall in cortisol levels in response to the TSST was observed. Given these results, we further examined a conditional model that included social anxiety and PEP.

### Conditional model: impact of social anxiety and PEP on cortisol recovery

We conducted a conditional model to examine the interaction between the SA composite and PEP predicting cortisol recovery. The model summary is provided in Table 2. PEP and social anxiety interactively predicted rate of recovery (*b* = −0.0005, *p* = 0.022). The effect size for this interaction was medium to large (Hedges' *g* = 0.710). Hence, simple slope analyses were performed with high (+1 *SD* above mean) and low (−1 *SD* below mean) conditional values of PEP and social anxiety.

**Table 2:** Modeling of social anxiety and PEP predicting salivary cortisol peak, activation, and recovery, controlling for the baseline levels, depression, and gender.

	***b***	***SE***	***t***	***P***
Intercept (Peak)	−1.424	0.038	37.569	<0.001
Activation slope	0.011	0.003	3.847	<0.001
Recovery slope	−0.009	0.001	9.286	<0.001
Baseline cortisol	0.577	0.099	5.849	<0.001
Depression	−0.005	0.003	1.594	0.112
Gender	−0.0002	0.078	0.003	0.998
PEP	0.006	0.013	0.455	0.652
PEP × Activation slope	0.00009	0.0009	0.093	0.926
PEP × Recovery slope	0.00009	0.00002	0.353	0.724
SA composite	0.011	0.046	0.231	0.819
SA composite × Activation slope	−0.002	0.003	0.530	0.597
SA composite × Recovery slope	−0.0010	0.0008	1.250	0.213
SA composite × PEP	0.003	0.013	0.203	0.840
SA composite × PEP × Activation slope	0.0005	0.0013	0.380	0.704
SA composite × PEP × Recovery slope	−0.0006	0.0002	2.301	0.022

*SA composite, social anxiety composite score (averaged Z score of social phobia scale and social interaction anxiety scale); PEP, post-event processing (assessed using thought sampling procedure)*.

**Figure 1:**
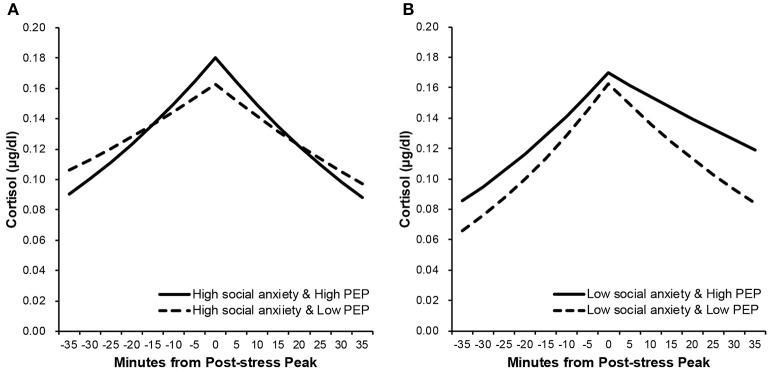
Estimated cortisol response trajectories before and after peak response to the TSST. Simple slopes are plotted at 1*SD* above and below means of PEP and social anxiety composite scores. The panel **(A)** shows slopes at high levels of social anxiety, and the panel **(B)** shows slopes at low levels of social anxiety. Values are back-transformed from box-transformation.

Figure 1 shows estimated cortisol response trajectories before and after peak response to the TSST at 1*SD* above and below means of PEP and SA composite scores. At low levels of social anxiety, recovery rate was slower at high levels of PEP (*b* = −0.006, *p* = 0.003) than at low levels of PEP (*b* = −0.011, *p* < 0.001). In addition, slope difference test (Robinson et al., 2013) confirmed that the difference between these slopes was statistically significant (*t* = 2.214, *p* = 0.030). At high levels of social anxiety, however, recovery rate was slower at low levels of PEP (*b* = −0.009, *p* < 0.001) than at high levels of PEP (*b* = −0.012, *p* < 0.001). The difference between these slopes was also statistically significant (*t* = 2.384, *p* = 0.019). For activation slope and absolute peak response, no significant effect of PEP and social anxiety was observed.

## Discussion

The present study investigated the effect of PEP on impaired cortisol recovery following social evaluative stressors. We hypothesized that individuals with higher levels of social anxiety and more frequent PEP would exhibit impaired cortisol recovery compared to individuals with higher levels of social anxiety and less frequent PEP. Results indicated that PEP and social anxiety interactively predicted cortisol recovery after the peak response. However, the hypothesized effect of PEP was observed at low levels of social anxiety but not at high levels of social anxiety; we predicted that the effect of PEP would be most robust among individuals with greater social anxiety symptoms. Thus, our hypotheses were not clearly supported.

The observed effect of PEP among individuals with low levels of social anxiety itself is not surprising, given previous findings that show the effect of PEP on cortisol recovery in healthy undergraduates (Zoccola et al., 2008). However, no prominent effect of PEP among individuals with high levels of social anxiety was inconsistent with our initial hypothesis. Rather, PEP was associated with faster recovery at high levels of social anxiety. This unexpected finding could be partially interpreted in terms of a model of protective inhibition (protective inhibition of self-regulation and motivation; PRISM; Tops et al., 2015). Protective inhibition refers to a mechanism of the nervous system to protect itself against an overload of stimulation. In this perspective, for individuals with high levels of social anxiety, physiological disengagement may serve to protect against negative consequences of unmanageably high emotional arousal. In addition, in PRISM framework, the relationship between social threat and physiological disengagement is moderated by potential motivation (i.e., the personal importance associated with engaging in and mobilizing physiological resources to actively cope with the situation). In the present study, the TSST may have imposed acute personally-important social evaluative threat that increased cortisol responses, and subsequent engagement in PEP may have further caused prolonged emotional arousal. On the other hand, the acute personal importance attributed to active coping during subsequent cognitive task performance may not have been necessarily high. Thus, the combination of high emotional arousal caused by PEP and relatively low personal importance to active coping may have resulted in physiological disengagement (i.e., faster cortisol recovery) in individuals with high levels of social anxiety. To further support this hypothesis, future studies should employ an assessment of personal importance of active coping for the situation or an experimental manipulation of it.

Another possibility is that heterogeneity in cortisol reactivity among individuals with social anxiety confounded the levels of PEP. Although no significant effect of social anxiety and PEP on absolute peak response was observed, Figure 1 suggests that absolute peak response differs depending on levels of PEP at high levels of social anxiety. Indeed, some individuals with social anxiety symptoms are reported to exhibit blunted cortisol response toward social stressors (Furlan et al., 2001). Such blunted HPA-axis reactivity in highly socially anxious individuals could be interpreted as a result of repeated exposure to social stressors in daily life (e.g., Schommer et al., 2003; Shirotsuki et al., 2009). Lower peak responses generally lead to slower recovery (Lopez-Duran et al., 2015), which may explain why no clear effect of PEP was found at high levels of social anxiety. However, as our results are based on estimations by individuals with various levels of social anxiety, further examination in the socially anxious population is necessary.

Several study limitations should be noted. First, the present findings are based on a relatively small sample. Larger sample sizes are preferred for purposes of generalizability. Second, it is possible that our thought sampling procedure did not necessarily assess spontaneous PEP. In particular, although the thought sampling procedure in the present study is well-established and has been used to assess post-stress thought, it is still possible that cognitive tasks may have prevented PEP to some degree. Third, we did not collect body mass index data, which is known to impact neuroendocrine dysregulation (Champaneri et al., 2013). Additionally, although most female participants were in their late luteal or early follicular phase of their menstrual cycle and the influence of gender was controlled in the analysis, more rigorous control for the influence of sex hormones is needed. In addition, it would also be important to examine whether gender moderate the effect of PEP and social anxiety on cortisol recovery (e.g., Shull et al., 2016). Finally, the present results are derived from a non-clinical sample. Future replication of these relationships with an actual clinical, SAD sample is desired.

To conclude, the findings in the present study provide preliminary evidence that the effect of PEP on cortisol recovery following social stressors differs depending on levels of social anxiety. However, the robust effect of PEP was observed only at low levels of social anxiety, which is an unexpected finding. Future studies should re-examine the effect of PEP on cortisol recovery with a refinement in thought sampling procedure or an explicit PEP manipulation procedure, as well as consideration of personal importance associated with actively coping with the situation. Greater understanding of the role of PEP in cortisol recovery is an important goal for future studies, as it can help uncover biological bases of social anxiety, which may subsequently leads to tailored psychological and pharmacological interventions for social anxiety.

## Author contributions

SM, TS, and HS designed the experiment. HT provided advice on data acquisition and analysis. SM performed the experiments and analyzed the data. All authors discussed the results and contributed to the writing of the paper.

### Conflict of interest statement

The authors declare that the research was conducted in the absence of any commercial or financial relationships that could be construed as a potential conflict of interest.
