# Supplementary material for: Post-event Processing Predicts Impaired Cortisol Recovery Following Social Stressor: The Moderating Role of Social Anxiety
Source: Front Psychol. 2017 Oct 31;8:1919. doi: 10.3389/fpsyg.2017.01919 (PMC5671589; doi:10.3389/fpsyg.2017.01919)
Supplement: Supplementary file 1 [file DataSheet1.DOCX]

Supplementary Material

Post-event processing predicts impaired cortisol recovery following social stressor: The moderating role of social anxiety

Shunta Maeda*, Tomoya Sato, Hironori Shimada, and Hideki Tsumura

*** Correspondence:** Shunta Maeda: [maeda_s@suou.waseda.jp](mailto:maeda_s@suou.waseda.jp)

**
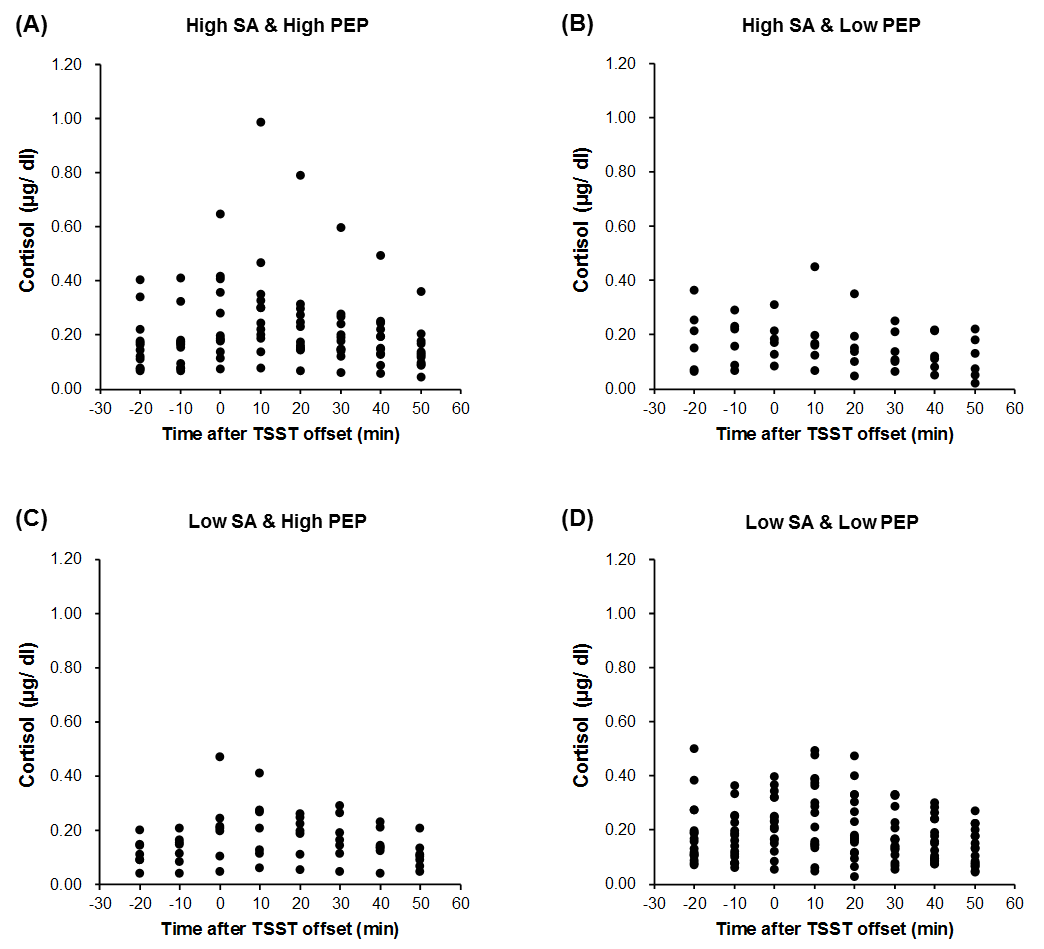
**

**Supplementary Figure 1.** The relationship between time and cortisol for high and low levels of social anxiety and PEP, based on median split. Panel (A) shows plots at high levels of social anxiety and high levels of PEP. Panel (B) shows plots at high levels of social anxiety and low levels of PEP. Panel (C) shows plots at low levels of social anxiety and high levels of PEP. Panel (D) shows plots at low levels of social anxiety and low levels of PEP.


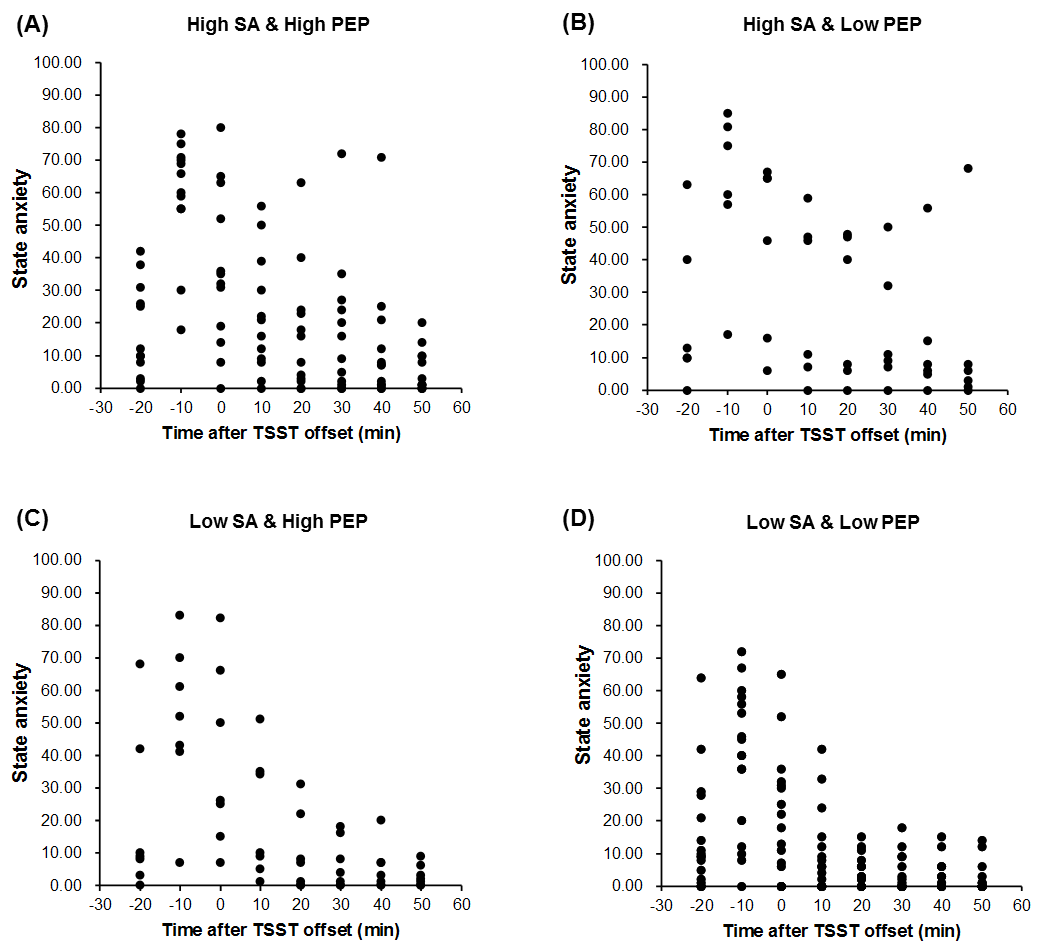


**Supplementary Figure 2.** The relationship between time and state anxiety for high and low levels of social anxiety and PEP, based on median split. Panel (A) shows plots at high levels of social anxiety and high levels of PEP. Panel (B) shows plots at high levels of social anxiety and low levels of PEP. Panel (C) shows plots at low levels of social anxiety and high levels of PEP. Panel (D) shows plots at low levels of social anxiety and low levels of PEP.

**Supplementary Table 1.**

Hierarchical regression analysis predicting task performance (number of error responses) throughout the Choice Reaction Task and the Working Memory Task.

| Predictors | Δ*R*^2^ |  | Δ*F* | β |  | *t* |
| --- | --- | --- | --- | --- | --- | --- |
| *Step 1* | .08 |  | 2.10 |  |  |  |
| SA composite |  |  |  | -.14 |  | 0.94 |
| PEP |  |  |  | .28 | ^†^ | 1.86 |
| *Step 2* | .07 |  | 2.16 |  |  |  |
| SA composite |  |  |  | -.33 |  | 1.65 |
| PEP |  |  |  | .50 | ^*^ | 2.12 |
| SA composite x PEP |  |  |  | -.36 |  | 1.47 |
|  |  |  |  |  |  |  |
| Total *R*^2^ | .15 | ^†^ |  |  |  |  |

*Note*. SA composite = social anxiety composite score (averaged *Z* score of social phobia scale and social interaction anxiety scale); PEP = post-event processing (assessed by thought sampling procedure); ^†^ *p* < .10, ^*^ *p* < .05.
